# The Propagation of Cattle Tuberculosis by Fecal Matter

**Published:** 1897-02

**Authors:** 


### ABSTRACTS FROM FOREIGN JOURNALS.

UNDER THE DIRECTION OF

J. PRESTON HOSKINS,

PRINCETON, N. J.

THE PROPAGATION OF CATTLE TUBERCULOSIS BY FECAL MATTER. Cadéac and Bonmay have already demonstrated experimentally that the bacillus of tuberculosis retains all its virulence after traversing the digestive tract of the dog. The dog at the same time that it becomes infected by ingestion can also become a vehicle for transmission of the virus of this disease. Messrs. Cadéac and Bonmay have made like experiments upon cattle, and affirm that animals of the group which have been infected with tuberculous products expel with their excrement bacilli whose virulency they have proved by inoculating rodents. The fecal matter of tuberculous cattle thus presents itself as one of the agents of infection, whether the animal has ulcerous lesions in the digestive tube or swallows the products of suppuration from the pulmonary lesions.—*Annales de Méd. Vét.*, March, 1896.

